# Role of additives and solvents in the synthesis of chiral isoreticular MOF-74 topologies†

**DOI:** 10.1039/d1dt01945g

**Published:** 2021-07-21

**Authors:** Andreea Gheorghe, Suzanne Reus, Mark Koenis, David Dubbeldam, Sander Woutersen, Stefania Tanase

**Affiliations:** Van‘t Hoff Institute for Molecular Sciences, University of Amsterdam Science Park 904 1098 XH Amsterdam The Netherlands s.grecea@uv.nl

## Abstract

Chiral induction is a simple and inexpensive approach to synthesise chiral metal–organic frameworks, even when using achiral building-blocks. The challenge lies in selecting the proper chiral inductor. This can only be achieved upon understanding the mechanism behind the chirality transfer from the chiral guest to the achiral MOF. In this work, the role of two types of chiral additives and different solvents was investigated in the crystallization of isoreticular MOF-74. We show that pyrrolidone-based solvents can interact with the framework walls and influence the thermal stability of the MOF. The role of the different chiral additives is related to the strength of their interaction with the MOF. Unlike cinchona alkaloids that have weak interactions with the framework, l- or d-*trans*-4-hydroxyproline (l- or d-Hyp) can strongly bind to the Zn^2+^ metal centres and cause the twisting of the organic linker. Moreover, l- and d-Hyp additives can affect the IRMOF-74 nucleation process depending on their concentration and handedness.

## Abstract 2

Strongly interacting chiral additives play a complex role during the crystallisation of chiral isoreticular MOF-74. They can coordinate to the open sites of the metal ions and induce strain on the framework, leading to a local twisting of the organic linker.

## Graphical abstract

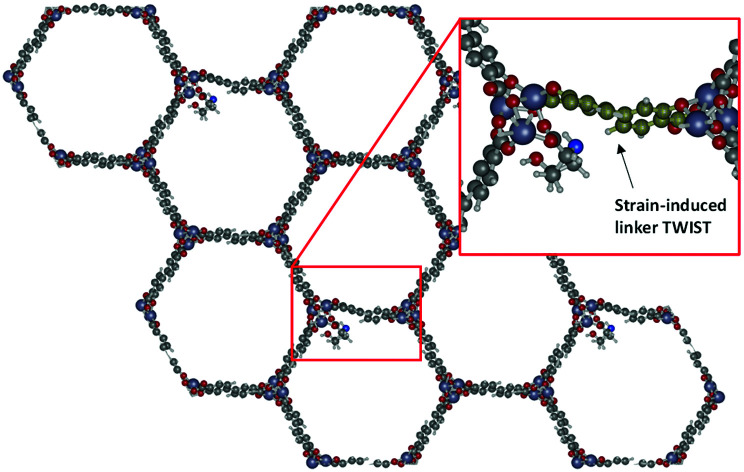

## Introduction

Chirality plays a key role in science and is an essential molecular feature of life on Earth.^1,2^ The chiral structure of biomacromolecules originates from asymmetric conformations which commonly include an asymmetric carbon or other chiral centers.^3^ In crystalline molecular assemblies, the chirality arises from the intrinsic asymmetry of the molecular building-blocks or from their assembly in the solid molecular structure. Therefore, understanding the chirality induction process is of utmost importance for the designed synthesis of molecular materials with controllable chirality. Chiral induction is regarded as a highly effective strategy for the synthesis of molecular assemblies with intrinsic chirality,^4^ in which a chiral molecule (commonly named chiral agent) is used as chiral seed to tune the overall homochiral assembly of achiral molecules.^5,6^ Using this approach, the synthesis of a homochiral MOF can be achieved by employing enantiopure guest molecules and tuning the interactions between the guest and the host framework to enable the chirality transfer from the intrinsic chirality of the guest to the conformational chirality of the MOF framework. For example, the chiral methyl-2-(benzylideneamino)propanoate (BPAM) was used as a chiral inductor in the homochiral spontaneous resolution of a silver-based MOF (Ag-MOF).^7^ Considering the single-crystal XRD studies, it was proposed that the chiral BPAM molecule coordinates to the Ag^+^ ions and then this interaction subsequently induces the initial chirality of Ag^+^.^7^ The Ag-MOF is built from an organic linker containing trisphenylamine moieties and the atropisomeric chirality of these moieties facilitates the chiral transfer between the Ag^+^ centres, leading to the symmetry breaking during the competitive crystallizations.^7^

The nucleation process of MOF-74 can be influenced by additives such as 1,2,4-triazole,^8^l-proline (l-Pro) and *cinchona* alkaloids.^9^ Particularly, we have shown that employing *cinchona* alkaloids as chiral inductors impacts the nucleation process of Zn-MOF-74.^9^ The alkaloids act as coordination modulators leading to different achiral MOFs (HIMS-74 and UTSA-74), depending on the synthesis solvent used. All MOFs have a porous structure with 1D open channels, which are smaller for UTSA-74^10^ and HIMS-74^9^ (*ca.* 8.8 Å) than for MOF-74 (*ca.* 11 Å). The smaller pore sizes of the MOFs as compared to the molecular size of the alkaloids prevented their accommodation as guests in the MOF's structure, therefore inhibiting the induction of chirality. Furthermore, Zn-UTSA-74 and Zn-HIMS-74 isomers have relatively low stability and convert to Zn-MOF-74 upon exposure to air or humidity.^9^ This drawback might be overcome by using MOFs with larger pore size and identical network topology. The isoreticular approach to synthesise MOFs with identical topologies is a promising methodology to overcome the pore's accessibility limitations.^11–13^ However, its challenges relate to retaining topology^14^ or interpenetration.^15^

**Fig. 1:**
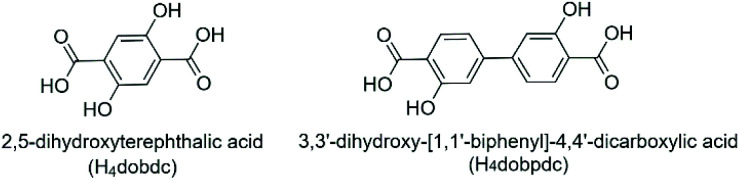
Molecular structure of the organic linkers of MOF-74 (left) and IRMOF-74 (right).

Zn-IRMOF-74 belongs to the Zn-MOF-74 isoreticular series and it has a honeycomb structure built from helical metal-oxo SBU chains connected through 3,3′-dihydroxy-[1,1′-biphenyl]-4,4′-dicarboxylic acid (H_4_dobpdc) linkers (Fig. 1, right).^11^ Similar to Zn-MOF-74, it belongs to the achiral space group *R*3̄, since the helical SBU chains have alternated handedness. The increased length of the organic linker leads to 1D channels with a diameter of *ca.* 17 Å, which would easily accommodate the *cinchona* alkaloids. Thus, using *cinchona* alkaloids may enable to tune the handedness of the SBUs and therefore induce chirality. Two types of interactions are possible between the guest alkaloid molecules and the Zn-IRMOF-74 host. On one hand, the alkaloid molecules could simply fill the Zn-IRMOF-74 pores and interact *via* non-covalent interactions with the pore walls, *e.g.* hydrogen bonding interactions or π–π stacking. On the other hand, a chiral transfer from the chiral alkaloids to Zn-IRMOF-74 can occur during nucleation due to its coordination to the Zn^2+^ ions of the SBUs *via* the amine or hydroxyl groups. This would then lead to a preferred orientation of the helical SBUs which ultimately changes the *P*/*M* ratio. Eventually, the framework formed would be built up from chains with either *P* or *M* configuration, based on the chirality of the alkaloid.

**Fig. 2:**
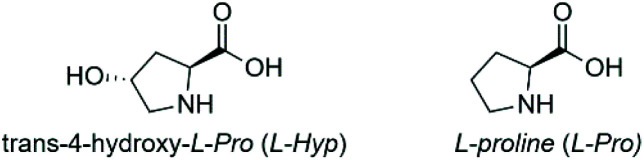
Molecular structure of proline chiral additives.

The interaction of chiral additives with the MOF framework is a key factor in chiral induction. Taking this into account, a different approach to improve pore accessibility is to use smaller chiral additives which have functional groups similar to those present in the organic linker of the guest MOF.^16,17^ It was shown that l-Pro affects the crystallisation process of Zn-MOF-74 and that the coordination of l-Pro to the Zn^2+^ metal ions of Zn-MOF-74 occurs *via* the carboxyl group.^9,18^ Therefore, it is relevant to study the role of *trans*-4-hydroxy-l-proline (l-Hyp) (Fig. 2, right) in the crystallisation of Zn-IRMOF-74. l-Hyp is a chiral proline derivative with carboxyl and hydroxyl functional groups that are also present in the H_4_dobpdc linker. l-Hyp can coordinate *via* the hydroxyl group to the open metal site of Zn-IRMOF-74 since it is less sterically hindered as compared to the carboxyl group. This type of binding would enable the formation of chiral MOF-74 as very promising candidate for applications in asymmetric catalysis due to the availability of both carboxyl and amine functional groups.^19–21^ Our studies aimed at determining the role of *cinchona* alkaloids and 4-hydroxyproline chiral additives and the effect of the synthesis solvent in the synthesis of Zn-IRMOF-74 topologies, with the ultimate goal to gain insight into the mechanism governing the chiral induction process.

## Results and discussion

### The role of the solvents

**Fig. 3:**
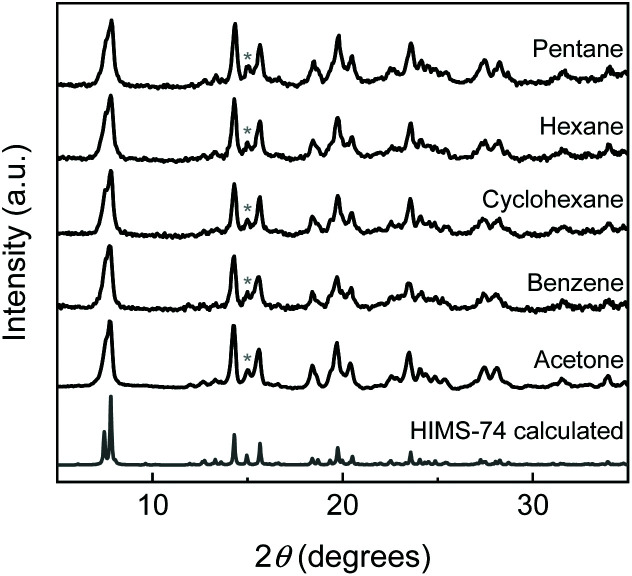
PXRD pattern of the simulated Zn-HIMS-74 (grey), and Zn-HIMS-74 samples recovered after immersing in acetone and different apolar solvents.

**Fig. 4:**
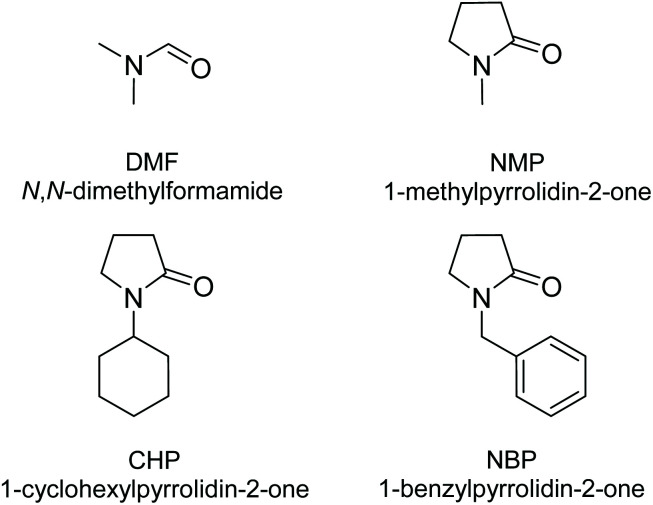
Molecular structure of the solvents employed in the crystallisation of Zn-IRMOF-74.

It is well-known that solvents influence the MOF's crystallisation process in terms of product formation^22–25^ and crystallinity.^24^ They are usually incorporated in the pores of as-synthesised MOFs as space filling free guest molecules as well as coordinated to the metal ions.^9,20^ Earlier studies^9^ showed that the synthesis solvent employed for the crystallisation of Zn-MOF-74 in the presence of alkaloids influenced the coordination environment of the Zn^2+^ ions, leading to different supramolecular isomers.^9^ For example, *N*-methyl-2-pyrrolidone (NMP) is a critical solvent for stabilizing the Zn-HIMS-74 framework. Removing NMP under thermal treatment leads to the collapse of the Zn-HIMS-74 framework whilst its replacement *via* solvent exchange procedures using coordinating polar solvents (methanol, ethanol, THF) facilitates the transformation of Zn-HIMS-74 to Zn-UTSA-74.^9^ Zn-HIMS-74 framework is retained upon its immersion in acetone or non-polar solvents, including pentane, hexane, cyclohexane, benzene, as indicated by PXRD studies (Fig. 3). The change of packing orientation mediated by the solvents is the predominant factor responsible for the molecular transformation of Zn-HIMS-74 to Zn-UTSA-74. Solvent molecules participate in the molecular stacking arrays in different ways and they prefer to interact with molecular domains sharing a similar polarity. For example, NMP molecules pack close to the aromatic rings of the organic linkers and the internal shearing stress created by NMP in the molecular structure of MOF-5 led to the stabilisation of a chiral framework.^26,27^ Based on these observations, the aim was to study the role of different pyrrolidone-based solvents in the stabilisation of Zn-IRMOF-74 framework. DMF was used as a reference and the series of pyrrolidone-based solvents included NMP, 1-cyclohexylpyrrolidin-2-one (CHP) and 1-benzylpyrrolidin-2-one (NBP) (Fig. 4). We expected that NBP would interact strongly with the Zn-IRMOF-74 framework though π–π stacking, therefore influencing significantly the crystallisation process.

**Fig. 5:**
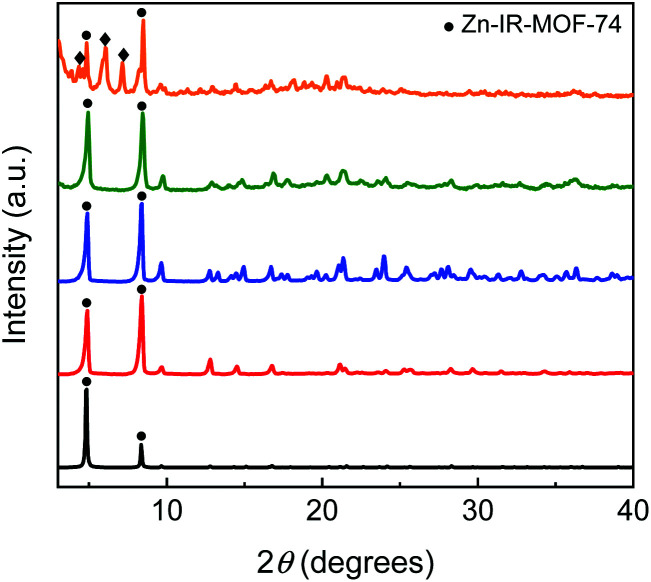
The PXRD patterns of Zn-IRMOF-74 synthesised in CHP (orange), NBP (green), NMP (blue), and DMF (red). The simulated Zn-IRMOF-74 calculated pattern (black) is used as ref. 27.

The reaction of Zn(NO_3_)_2_·6H_2_O with H_4_dobpdc in a ratio of 3 to 1 and using different synthesis solvents (DMF, NMP, CHP, and NBP) led to crystalline materials. Their PXRD patterns show the characteristic peaks of Zn-IRMOF-74.^27^ Nevertheless, the PXRD pattern of the material obtained in CHP shows additional peaks at 2*θ* = 4.4, 6.1, 7.2, indicating the simultaneous formation of other crystalline phases (Fig. 5). This could be due to the high flexibility of CHP arising from different cyclohexane conformations that might influence the product formation due to the different interaction with the secondary building-blocks. All other solvents employed do not affect the Zn-IRMOF-74 topology. However, the crystallinity of the Zn-IRMOF-74 materials seems to vary slightly, depending on the solvent used. The solvent also influences the morphology of the Zn-IRMOF-74 which crystallises as needles in DMF and NBP and spheres in NMP (observations using the polarised optical microscope as indicated in Fig. S2†). The impact of the solvent on the stability of Zn-IRMOF-74 was demonstrated using TGA-DSC analysis. Fig. S3† shows a shift of the DSC exothermic peak attributed to the framework decomposition. The shift to higher temperatures indicates an increase in the framework stability due to the solvent–solvent and MOF-solvent interactions in the order DMF < NMP < NBP. Using pyrrolidone-based solvents leads to more thermally stable frameworks, especially in the case of NBP that has aromatic rings able interact with the MOF walls.

### The role of chiral additives

Chiral additives influence the nucleation process of Zn-MOF-74.^9^ The presence of l-proline (l-Pro), a relatively small chiral additive, prevents the crystallisation of Zn-MOF-74. *Cinchona* alkaloid additives, *i.e.* (+)-cinchonine and (−)-cinchonidine act as coordination modulators in the formation of the SBUs and lead to supramolecular isomers.^9^ The alkaloids can interact as guest molecules *via* non-covalent interactions or coordinate to the Zn^2+^ ions. Since the synthesis solvent influences the stability and crystallinity of Zn-IRMOF-74, subsequent studies focused on the role of (−)-cinchonidine using DMF and NMP as solvents. Bulkier solvents were not studied due to limitations arising from the confined pores and the size of the chiral additive.

The hydrothermal synthesis of Zn-IRMOF-74 was performed by reacting Zn(NO_3_)_2_·6H_2_O, H_4_dobpdc (Zn^2+ ^: H_4_dobpdc molar ratio of 3 : 1) and (−)-cinchonidine (3 equiv.). The crystalline materials formed were characterised to determine the sample purity and chirality. PXRD analysis shows that irrespective of the alkaloid and synthesis solvent employed, the Zn-IRMOF-74 topology is obtained (Fig. S4,† left). The FTIR spectra displayed only the characteristic absorption bands of Zn-IRMOF-74 upon comparison with the FTIR spectrum of the as-synthesised Zn-IRMOF-74 in DMF and NMP (Fig. S4,† right). It indicates that the alkaloid is not present in the pores of the MOF. Because of the similar results in terms of topology and sample purity, only Zn-IRMOF-74 obtained in the presence of (−)-cinchonidine and using NMP as solvent was further investigated.

The TGA-DSC analysis of the Zn-IRMOF-74 synthesised in NMP and in the absence of the alkaloid shows an exothermic DSC peak at 423 °C (Fig. S3†). For the sample synthesised in the presence of (−)-cinchonidine, the large exothermic DSC peak at *ca*. 400 °C corresponds to the framework decomposition and an additional small exothermic DSC peak is present at 423 °C (Fig. S5†). This indicates that the chiral additive influences the stability of the framework and might even be present in the framework in small amounts, as indicated by the *ca.* 3% difference in the residual mass between the MOF obtained with and without alkaloid (Fig. S3 and S5†). Indeed, the analysis of the as-synthesised Zn-IRMOF-74 in the mother liquid using a polarised optical microscope reveals the typical needle-shaped crystals of Zn-IRMOF-74 and the excess alkaloid as aggregates in the turbid solution (Fig. S6†).

The presence of the alkaloid in small amount in the pores of Zn-IRMOF-74 does not prevent the induction of chirality, which could still occur during the nucleation process. To verify the presence of chirality, vibrational circular dichroism (VCD) measurements were performed for the samples obtained in the presence of (+)-cinchonine or (−)-cinchonidine, and using NMP or DMF as solvents (Fig. S7†). The Zn-IRMOF-74 topology of these samples was confirmed by the PXRD studies (Fig. S8†). Although VCD spectra of the possibly enantiomeric MOFs were recorded, yet no significant VCD signals were obtained (Fig. S7†). Thus, even though the size of the alkaloid allows its diffusion in the open channels of Zn-IRMOF-74 with *ca.* 17 Å, the induction of chirality is not achieved. This is most likely due to the low affinity of the MOF towards the alkaloids since only small amounts of alkaloid diffused in the framework. It is in agreement with earlier studies showing that relatively strong interactions of the additive with the building-blocks of the framework are required to attain chiral induction.^28^

Our previous studies showed that post-synthetic modification of Zn-MOF-74 leads to coordination of l-Pro to Zn^2+^ metal ions though its carboxyl functional group. Adding l-Pro in the crystallisation of Zn-MOF-74 is also known to influence the nucleation process.^9^ Long *et al.*^29^ used l-Pro as a chiral seed for the crystallisation of isoreticular Mg-MOF-74 frameworks built from 3,3′-dihydroxy-[1,1′-biphenyl]-4,4′-dicarboxylic acid linkers.^29^ A such MOF consists of a racemic mixture of chiral subdomains that contain helices of the same orientation, unlike Zn-MOF-74 which has alternated *P* and *M* helices in the same domain.^29^ The concentration of the chiral additive is another important parameter for chiral induction. The crystallization of Zn-MOF-74 is affected by the addition of l-Pro, an effect also observed by Long *et al.*^29^ in the synthesis of Mg-IRMOF-74. Increasing the concentration of l-Pro influenced the MOF yield and eventually fully hindered the crystallisation.^29^

Since the mechanism by which chiral induction occurs is not reported, subsequent studies focused on the induction of chirality in Zn-IRMOF-74 using proline-based derivatives as chiral inductors. We have selected *trans*-4-hydroxy-l-proline (l-Hyp) (Fig. 2, right) because it has carboxyl and hydroxyl functional groups, similar to those present in the H_4_dopbdc linker. This is important to ensure a strong affinity between chiral additive and the porous molecular framework. Unlike l-Pro, it is expected that l-Hyp will coordinate to the Zn^2+^ ions *via* the hydroxyl group. The role of the additive's concentration on the nucleation process was studied by synthesising Zn-IRMOF-74 in the presence of 1 or 3 equiv. of l-Hyp. Both NMP and NBP were used as solvents because l-Hyp has significantly lower size (5.9 Å × 2.0 Å × 3.3 Å) as compared to the *cinchona* alkaloids (*e.g.* 11.8 × 9.3 × 7.3 Å^3^ for (+)-cinchonine). We expected that pyrrolidone-based solvents will create a shear tension in the Zn-IRMOF-74 framework *via* solvent–solvent interactions, especially in the case of NBP which can interact with the framework's walls though π–π stacking and in turn lead to the induction of chirality.

**Scheme 1:**
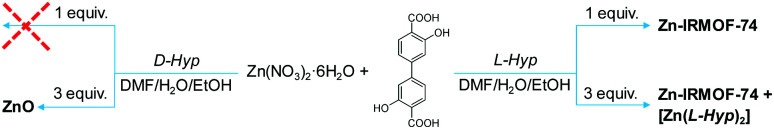
The synthesis of Zn-IRMOF-74 in the presence of l- or d-Hyp using a Zn^2+^ : H_4_dobdc molar ratio of 3 : 1.

**Fig. 6:**
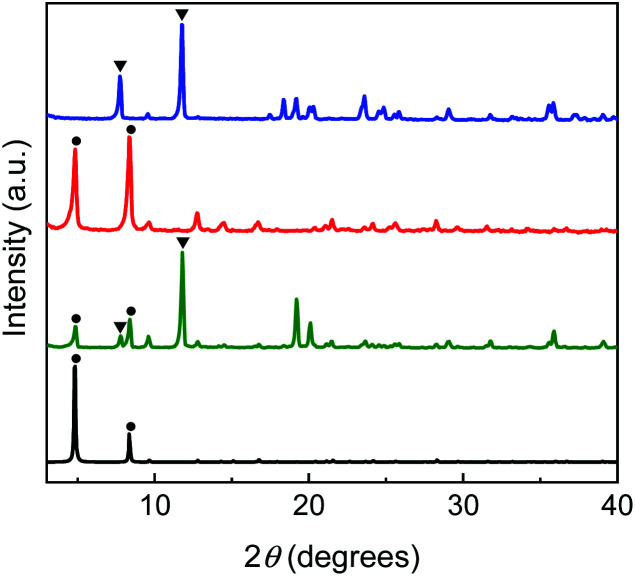
PXRD patterns of Zn-IRMOF-74 obtained in DMF, in the presence of l-Hyp in a 1 : 1 : 1 (blue), 3 : 1 : 1 (red), 3 : 1 : 3 (green) Zn^2+ ^: H_4_dobpdc^ ^: l-Hyp molar ratios, and the simulated PXRD pattern for Zn-IRMOF-74 (black).^27^ The circles indicate the Zn-IRMOF-74 phase and the triangles correspond to the [Zn(l-Hyp)_2_] phase.

The hydrothermal synthesis of Zn-IRMOF-74 was performed by reacting Zn(NO_3_)_2_·6H_2_O, H_4_dobpdc (Zn^2+ ^: H_4_dobpdc molar ratio of 3 : 1) and 4-hydroxyproline (1 or 3 equiv.) using DMF, NMP and NBP as solvents. Scheme 1 summarises the results when using DMF as main solvent. All materials obtained in DMF were characterised using PXRD (Fig. 6). The PXRD pattern of the crystalline material obtained by using a large amount of l-Hyp (Zn^2+ ^: H_4_dobdc : l-Hyp molar ratio equal to 3 : 1 : 3) shows the characteristic peaks corresponding to the Zn-IRMOF-74 phase and some additional peaks at 2*θ* = 7.8° and 11.8°, indicating the formation of another crystalline phase. Alterations to the Zn-IRMOF-74 network topology were not expected. By employing l-Pro as synthesis modulator^30^ or for functionalisation,^31^ the coordination of l-Pro to the open metal sites does not lead to changes in the overall structural topology and subsequently, the PXRD patterns are not altered. The Zn^2+ ^: H_4_dobpdc molar ratio equal to 3 : 1 favours the formation of Zn-IRMOF-74.^32^ Therefore, a Zn^2+ ^: H_4_dobpdc : l-Hyp molar ratio equal to 1 : 1 : 1 is expected to give insight into the crystallisation process based on the competition between H_4_dobpdc and l-Hyp for the coordination to the Zn^2+^ ions. The PXRD pattern of the crystalline material obtained for the 1 : 1 : 1 Zn^2+ ^: H_4_dobpdc : l-Hyp molar ratio contains only the peaks at 2*θ* = 7.8° and 11.8° (Fig. 6), confirming that the hydrothermal reaction using a Zn^2+ ^: H_4_dobpdc : l-Hyp molar ratio equal to 3 : 1 : 3 leads to a mixture of Zn-IRMOF-74 and a new crystalline phase.

**Fig. 7:**
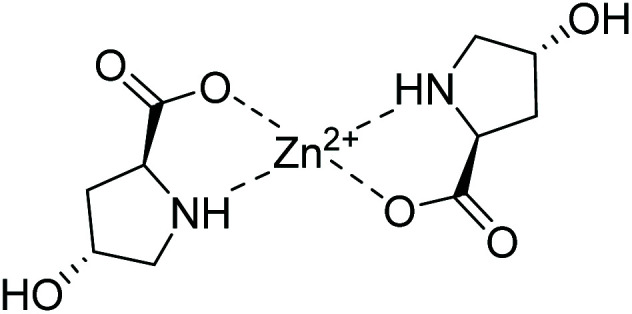
Proposed molecular structure of [Zn(l-Hyp)_2_].

It is well-known that the [Zn(l-Pro)_2_] complex forms under basic conditions at a pH = 13.6.^33^ A similar complex of Zn^2+^ with l-Hyp can also form during the hydrothermal synthesis of Zn-IRMOF-74. DMF has a pH of 6.3, however, during hydrothermal synthesis it undergoes hydrolysis to produce formic acid and dimethylamine.^34–36^ When DMF has been hydrolysed and it is in equilibrium with its degradation products, the pH reaches a value of 11.33,^35^ therefore it can facilitate the formation of the [Zn(l-Hyp)_2_] complex. Fig. 7 shows the proposed structure of [Zn(l-Hyp)_2_].

Based on the above observations, [Zn(l-Hyp)_2_] was synthesised separately by reacting Zn(CH_3_COO)_2_·2H_2_O (1 equiv.) with l-Hyp (2.3 equiv.). To accelerate the formation of the bis-chelate complex, NaOH was used to promote the deprotonation of the carboxylate group. The reaction was performed using a slight excess of l-Hyp to ensure the complexation of all the metal ions present in solution. The FTIR spectra confirm the carboxyl deprotonation as the band at 2730 cm^−1^ assigned to the carboxyl O–H stretch disappears upon binding of l-Hyp to the Zn^2+^ ions (Fig. S9†). TGA-DSC (Fig. S10†) of [Zn(l-Hyp)_2_] shows an initial weight loss of 2.3% that corresponds to a small broad DSC endothermic peak around 80 °C. It indicates the presence of non-coordinated water molecules, in agreement with the [Zn(l-Hyp)_2_]·0.5H_2_O formulation reported previously.^37^

A particularity of this complex relates to the presence of two *ν*_OH_ red shifted at 3303 and 3340 cm^−1^ due to the hydrogen bonding interactions established between the hydroxyl group and the carbonyl of adjacent ligand molecules or to a water molecule as in the case of [Pd(l-Hyp)_2_]·3H_2_O.^38^ Additional hydrogen bonding of the water to another carboxylate oxygen supports the two *ν*_OH_ red shifted bands. The decomposition of the complex is observed between 370–530 °C, with the corresponding exothermic effect on the DSC curve at *ca.* 465 °C. The formation of the bis-chelate complex is also supported by the mass spectrometry studies. Due to the presence of intermolecular hydrogen bonding interactions, dimers or trimers were also detected which indicate the formation of extended structures. To further support such interactions, single-crystal XRD analysis is needed since formation of such species could also be due to the sample ionisation during mass analysis. Nevertheless, attempts to obtain good quality single-crystals were unsuccessful.

The PXRD pattern of [Zn(l-Hyp)_2_] displays diffraction peaks at 2*θ* = 7.8° and 11.8° which enabled a facile matching with the new crystalline phase obtained in DMF during the crystallisation of Zn-IRMOF-74 using the Zn^2+ ^: H_4_dobdc : l-Hyp molar ratios equal to 3 : 1 : 3 (Fig. S11† and Fig. 6). This confirms that using excess of chiral additive during the crystallisation of Zn-IRMOF-74 leads to the formation of [Zn(l-Hyp)_2_] which in turn affects the yield of Zn-IRMOF-74. A low reaction yield when using high concentrations of chiral additives was also reported by Long *et al.*^29^

**Fig. 8:**
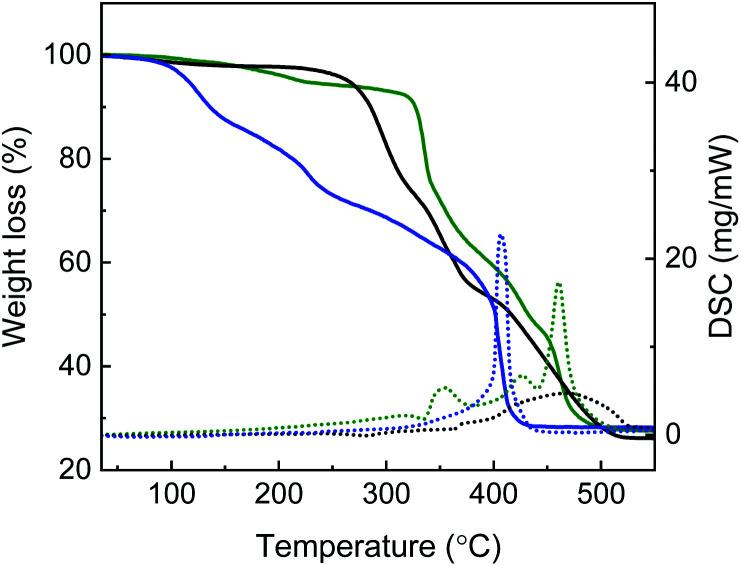
TGA-DSC curves of [Zn(l-Hyp)_2_] (black), Zn-IRMOF-74 synthesised in DMF (blue) and Zn-IRMOF-74 synthesised using a Zn2^+ ^: H_4_dobpdc^ ^: l-Hyp ratio equal to 3 : 1 : 3 (green).

TGA-DSC analysis was carried out on the sample prepared using Zn^2+ ^: H_4_dobpdc^ ^: l-Hyp in a 3 : 1 : 3 molar ratio, the [Zn(l-Hyp)_2_] complex, and Zn-IRMOF-74 synthesised in DMF (Fig. 8). The MOF prepared in DMF shows an initial 12% weight loss at around 120 °C, corresponding to the removal of H_2_O molecules. The second step of 15% mass loss observed around 230 °C corresponds to the removal of DMF. The decomposition of the framework is indicated by the sharp exothermic peak observed in the DSC at *ca*. 405 °C. The mixed phase sample shows a different TGA curve, with an initial weight loss of *ca*. 1.7% corresponding to the elimination of H_2_O molecules between 35 and 150 °C, followed by a 4% weight loss between 150 and 250 °C due to the removal of DMF molecules.

The presence of l-Hyp both as a guest molecule stabilised *via* weak interactions and as coordinated to the open metal sites of Zn-IRMOF-74 is indicated by the additional DSC peaks in the 300–400 °C range. The endothermic peak around 335 °C corresponds to the decomposition of weakly bound l-Hyp that has a melting point of 274 °C. The presence of coordinated l-Hyp is supported by the exothermic peak at *ca.* 355 °C. The decomposition of the organic linker and the collapse of the framework occurs at *ca.* 425 °C and 460 °C, respectively. A shift to higher values of the decomposition temperature of Zn-IRMOF-74 containing the coordinated chiral additive indicates an increased stability of the molecular framework.

Because of the formation of both l-Hyp-modified Zn-IRMOF-74 MOF and [Zn(l-Hyp)_2_], the chirality of the Zn-IRMOF-74 derivative prepared using a large concentration of l-Hyp could not be determined. A significant shift of the vibrational circular dichroism (VCD) signals would occur upon the coordination of l-Hyp to the Zn^2+^ metal ions of Zn-IRMOF-74 as calculated for the [Zn(l-Hyp)_2_] complex and free l-Hyp (Fig. S12†). It could be that the additive acts as an auxiliary linker because of the increased concentration employed. This would explain the appearance of the PXRD peaks characteristic for Zn-IRMOF-74 since the overall topology is retained. Using a lower concentration of l-Hyp is expected to prevent the formation of [Zn(l-Hyp)_2_]. Indeed, the PXRD of the sample prepared using a Zn^2+^ : H_4_dobpdc : l-Hyp in a 3 : 1 : 1 molar ratio showed only the characteristic peaks of Zn-IRMOF-74 (Fig. 6). Introducing chirality to the framework is not expected to change the position of the atoms since the distance between atomic planes would remain similar. This is also observed for the PXRD of the chiral Mg-IRMOF-74.^29^ The TGA-DSC of this sample was also measured (Fig. S13†). The sample shows less porosity as compared to Zn-IRMOF-74 synthesised in DMF. It is based on the 15% weight loss observed in the range 35–250 °C as compared to the 27% weight loss observed for Zn-IRMOF-74 synthesised in DMF. The initial weight loss of *ca.* 2% in the range 35–150 °C corresponds to the removal of H_2_O molecules. The DSC endothermic peak at 220 °C is attributed to the removal of DMF molecules and it relates on the TGA curve to the *ca.* 13% weight loss. Additionally, a small endothermic effect at *ca.* 280 °C is observed that might correspond to the l-Hyp present on the surface or within the MOF channels. This indicates that when using a small amount of chiral additive, the additive acts as a chiral inductor since it should leave the chiral MOF.^4^ Two sharp exothermic peaks at *ca.* 410 and 415 °C correspond to the framework decomposition, also indicating an increased framework stability.

The chirality of the samples could not be determined since the synthesis of Zn-IRMOF-74 in the presence of *trans*-4-hydroxy-d-proline (d-Hyp) was not possible. It could be that the interaction of the chiral additive with the Zn^2+^ ions during the hydrothermal reaction affects the MOF nucleation process. This result indicates that the handedness of the chiral additive can influence its interaction with the molecular framework and thereby influencing the nucleation process. Unfortunately, crystallisation of Zn-IRMOF-74 in the presence of a high concentration of d-Hyp (Zn^2+ ^: H_4_dobpdc^ ^: d-Hyp molar ratio equal to 3 : 1 : 3) was not successful and only the formation of ZnO was observed. Notably, the synthesis of [Zn(d-Hyp)_2_] requires a much longer reaction time (16 h) as opposed to the synthesis of the [Zn(l-Hyp)_2_] complex which forms in less than 1 h (Fig. S11†). Therefore, we can conclude that the synthesis of Zn-IRMOF-74 in DMF in the presence of l- or d-Hyp is dependent on both concentration and handedness of the chiral additive.

**Fig. 9:**
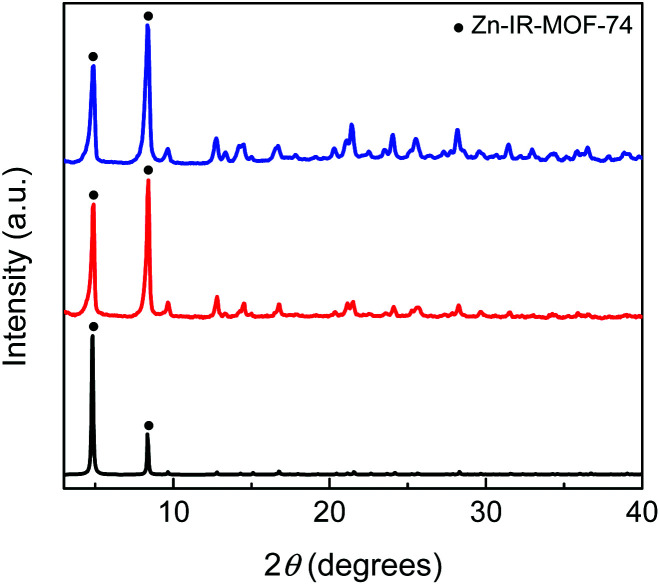
PXRD patterns of Zn-IRMOF-74 obtained in NMP using a 3 : 1 : 1 ratio Zn^2+ ^: H_4_dobpdc^ ^: Hyp molar ratio using l-Hyp (blue) and d-Hyp (red), and the simulated PXRD pattern for Zn-IRMOF-74 (black).^27^ The circles indicate the IRMOF-74 phase.

Subsequent studies aimed at using pyrrolidine-based solvents for the synthesis of chiral Zn-IRMOF-74 in the presence of Hyp. It was expected that the solvent–solvent interactions would induce an internal shearing stress that might stabilise a chiral Zn-IRMOF-74 framework. PXRD analysis of Zn-IRMOF-74 obtained in the presence of l-Hyp using NMP as solvent showed a different crystallisation process as compared with the one performed in DMF (Fig. 6 and Fig. 9). The synthesis performed in NMP with either l- or d-Hyp led to solid crystalline materials only for the 3 : 1 : 1 molar ratio. Neither formation of Zn-IRMOF-74 or [Zn(l-Hyp)_2_] were observed for the synthesis using a 3 : 1 : 3 Zn^2+ ^: H_4_dobpdc^ ^: l-Hyp molar ratio. Moreover, no solid material forms when using a 1 : 1 : 1 Zn^2+ ^: H_4_dobpdc^ ^: l-Hyp molar ratio. This could be due to the reduced basicity of the reaction medium as compared with the synthesis carried out in DMF. PXRD analysis of these materials confirmed the Zn-IRMOF-74 topology (Fig. 9). The TGA and DSC curves show no additional features as compared with those of the as-synthesised Zn-IRMOF-74 (Fig. S14†), implying that the chiral additive does not reside in the framework. This is confirmed by FTIR and VCD studies that support the absence of the chiral additive and the lack of chirality (Fig. S15†). Attempts to synthesise Zn-IRMOF-74 in NBP lead to changes in the MOF's topology (Fig. S16†), most likely due to the NBP interaction with the aromatic rings of the MOF linker which was initially envisioned to bring stability to the framework.

### Computational studies

Because the crystallisation of pure materials has proven to be challenging, computational methods were employed to determine the effect of Hyp on the Zn-IRMOF-74 synthesis. Our studies focused on calculating the binding energy of the chiral additive to the framework. As crystallisation of Zn-IRMOF-74 in the presence of l- and d-Hyp lead to surprisingly different results, we considered all four stereoisomers of 4-hydroxy-proline to analyse their interaction with the MOF host framework (Fig. S17†).

Changes in the reaction conditions during MOF synthesis, due to the hydrolysis of DMF, has influence on the structure of the additive. When the pH of the reaction gradually increases, the structure of Hyp changes. Thus, we have chosen to perform molecular simulations on three different possible structures of Hyp: zwitterion (**A**) corresponding to isoelectric pH as well as structures with deprotonated carboxyl (**B**) and with both deprotonated carboxyl and hydroxyl groups (**C**), corresponding to an increasingly basic pH (Fig. S18†).

For each molecule several starting orientations were used to calculate the binding energies between the unit cell of Zn-IRMOF-74 and structures **A**, **B** or **C** of the chiral additive (Table S1†). Previous results show a binding of l-Pro through the carboxylate group to Zn^2+^ ions of Zn-MOF-74.^18^ In this case, we expected that the coordination of 4-hydroxyproline to the Zn^2+^ ions of Zn-IRMOF-74 *via* the deprotonated hydroxyl group at a high pH to be the most favourable interaction because it is less sterically hindered as compared to the carboxyl group.

In the case of deprotonation of both carboxyl and hydroxyl groups, the chiral additive was not stable. Pyrrolidone ring opening takes place even before its coordination to the Zn^2+^ ions in Zn-IRMOF-74. Thus, the general trend observed in the calculations for all four stereoisomers of 4-hydroxyproline with either an **A** or **B** structure were noted (Table 1). Overall, calculations using a zwitterion form (**A**) show a higher binding energy which corresponds to a monodentate coordination *via* the carboxyl group (Fig. S19 & S20†). The Zn–O bond length increases for the *cis* isomers due to intramolecular interactions as the proton of the amine group is transferred to the uncoordinated carboxyl oxygen, which becomes protonated.

**Table 1:** The binding energies and their corresponding Zn–O bond lengths for coordinating *trans*-4-hydroxy-l-proline, *trans*-4-hydroxy-d-proline, *cis*-4-hydroxy-l-proline and *cis*-4-hydroxy-d-proline to Zn-IRMOF-74 *via* oxygen donor atoms

Chiral additive	Binding energy (kJ mol^−1^)		Zn–O bond length (Å)	
	**A**	**B**	**A**	**B**
*trans*-4-Hydroxy-l-proline (l-Hyp)	−254.64	−441.17	2.032	1.970, 2.054
*trans*-4-Hydroxy-d-proline (d-Hyp)	−276.79	−428.11	1.976	1.975, 2.037
*cis*-4-Hydroxy-l-proline	−249.02	−465.03	2.106	2.001, 2.112
*cis*-4-Hydroxy-d-proline	−199.87	−536.97	2.251	1.986, 1.979

The most favourable binding energy values correspond to the interaction with the MOF for the additive in the deprotonated carboxyl form (**B**). For almost all stereoisomers, a bidentate coordination *via* the carboxylate oxygens to two consecutive Zn^2+^ metal ions is preferred (Fig. S19 & S20†), with the lowest energy state corresponding to *cis*-4-hydroxy-d-proline (−536.97 kJ mol^−1^). A particular binding is the one for *cis*-4-hydroxy-l-proline where the donor oxygens are not both from the carboxylate group. One carboxyl oxygen and the hydroxyl oxygen are the ones that bind to two Zn^2+^ metal ions.

This is due to a strong intramolecular hydrogen bonding between the uncoordinated carboxyl oxygen and the hydroxyl group (Fig. S20 & Table S2†).

The bidentate coordination is achieved when the pentacoordinate environment around the Zn^2+^ metal ions suffers a spatial arrangements of its donor oxygens (Fig. S21†). In the pre-state, the square pyramid is formed of four oxygens in the same plane and one oxygen below. In the re-arrangement, one of the in-plane oxygens moves up in the plane to accommodate the 4-hydroxyproline molecule. The bond below the plane is broken, so the pentacoordination is retained.

**Fig. 10:**
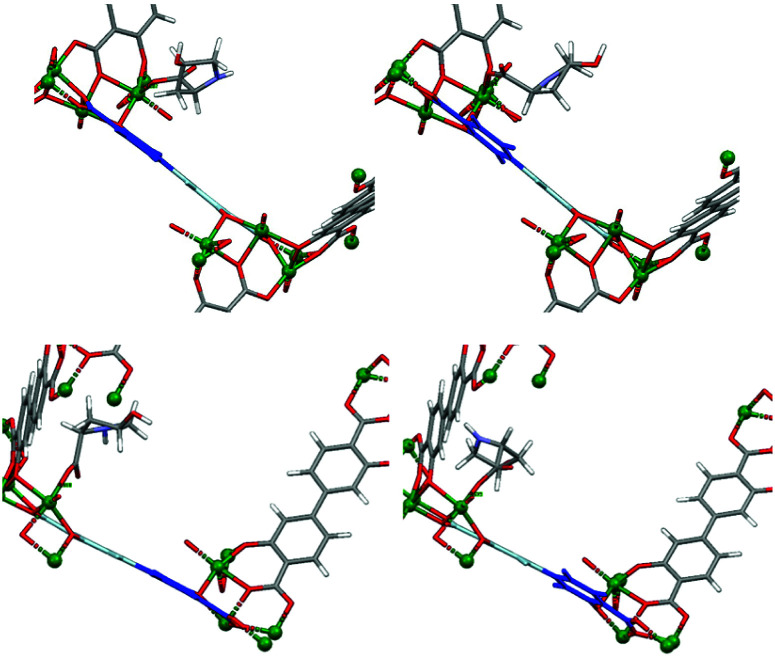
Strain upon coordination of deprotonated carboxyl d-Hyp (top) or l-Hyp (bottom), leading to twisting of the organic linker.

**Fig. 11:**
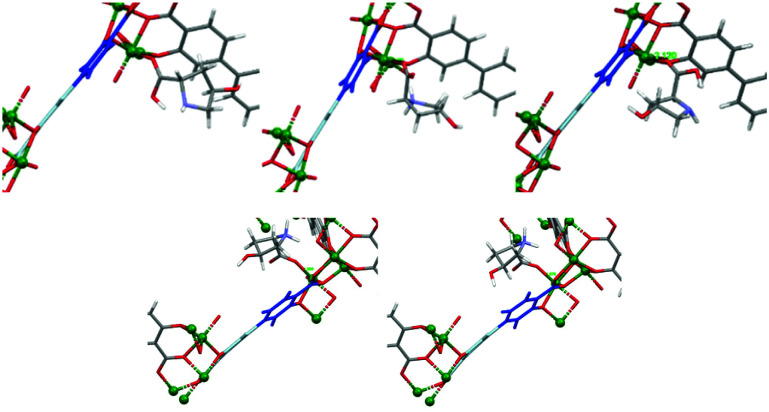
Strain upon coordination of zwitterion l-Hyp (top) or d-Hyp (bottom), with different orientations, leading to twisting of the organic linker.

In conclusion, the computational results show that all four stereoisomers of 4-hydroxyproline coordinate bidentate, since the energy states are lower compared to the monodentate binding. This is also consistent with the form of the additive in the reaction medium which increases in basicity and leads to a deprotonation of the carboxyl group. Another effect of the binding of the additive is the twisting of one of the aromatic rings of the organic linker upon coordination of the additive (Fig. 10 and 11).

## Experimental

### Materials and instrumentation

All chemicals were purchased from commercial suppliers and used without further purification. *trans*-4-Hydroxy-l-proline was procured from TCI with >99% purity and *trans*-4-hydroxy-d-proline was acquired from Sigma with 97% purity. Methanol used for the solvent exchange procedures was dried using molecular sieves with a diameter of 3 Å. Infrared spectra (4000–400 cm^−1^, resol. 1 cm^−1^) were recorded on a Varian 660 FTIR spectrometer using a KBr module. VCD spectra was recorded on the spinned solid samples, prepared as KBr pellets. VCD spectra were obtained on a Bruker Vertex 70 FTIR spectrometer equipped with a Bruker PMA50 VCD module. Powder XRD (3–60°, 2.5° min^−1^) measurements were carried out on a Rigaku Miniflex X-ray Diffractometer using Cu K-alpha radiation (*λ* = 1.5406 Å). Thermogravimetric analysis (35–500 °C, 5 K min^−1^) and differential scanning calorimetry (TGA-DSC) were performed using a NETZSCH Jupiter STA 449F3 instrument. The measurements were carried out under a combined flow of air (10 mL min^−1^) and protective argon (10 mL min^−1^). N_2_ adsorption isotherms were measured at 77 K on a Thermo Scientific Surfer instrument after stepwise evacuation. ^13^C NMR and ^1^H NMR spectra were recorded with a Bruker AMX 400.1 MHz spectrometer. DMSO-d_6_ was used as solvent, and the NMR spectra were referenced to the residual solvent signal. Electrospray ionization mass spectrometry (ESI-MS) was recorded on an AccuTOF LC, JMS-T100LP Mass spectrometer.

### General synthetic procedures

#### Synthesis of Zn-IRMOF-74 in different solvents

A modified literature procedure was used.^11^ Zn(NO_3_)_2_·6H_2_O (0.033 g, 0.109 mmol, 3 eq.) and H_4_dobpdc (0.010 g, 0.037 mmol, 1 eq.) and a solvent mixture of S : EtOH : H_2_O (20 : 1 : 1 v : v : v, 5.5 mL, S = DMF, NMP, CHP, NBP) was placed in a 10 mL Teflon screw–capped DuranTM Pyrex tube. The mixture was sonicated, then placed in a preheated oven and kept for 48 h at 120 °C. The reaction mixture was then cooled down to room temperature and the solid crystalline material was filtered and washed two times with synthesis solvent mixture, then it was dried in an oven at 80 °C for 12 h.

#### Synthesis of Zn-IRMOF-74 in the presence of cinchona alkaloids

A mixture of Zn(NO_3_)_2_·6H_2_O (0.033 g, 0.109 mmol, 3 eq.), H_4_dobpdc (0.010 g, 0.037 mmol, 1 eq.), *cinchona* alkaloid (0.032 g, 0.109 mmol, 3 eq.) and a solvent mixture of S : EtOH : H_2_O (20 : 1 : 1 v : v : v, 5.5 mL, S = DMF or NMP) was placed in a 20 mL Teflon screw–capped DuranTM Pyrex tube. The mixture was sonicated, then placed in a preheated oven and kept for 48 h at 120 °C. The reaction mixture was then cooled down to room temperature and the solid crystalline material was filtered and washed five times with the solvent mixture for the materials prepared in DMF : EtOH : H_2_O or three times with NMP and two times with acetone, for those synthesized in NMP : EtOH : H_2_O. Then, it was dried in an oven at 80 °C for 12 h.

#### Synthesis of Zn-IRMOF-74 in the presence of *trans*-4-hydroxy-l-proline

Zn(NO_3_)_2_·6H_2_O (0.011 g, 0.036 mmol, 1 eq.), H_4_dobpdc (0.010 g, 0.036 mmol, 1 eq.) and *trans*-4-hydroxy-l-proline (l-Hyp) (0.014 g, 0.036 mmol, 1 eq.) was dissolved in a solvent mixture of S : EtOH : H_2_O (20 : 1 : 1 v : v : v, 6 mL, S = DMF, NMP, NBP). The 10 mL Teflon screw–capped DuranTM Pyrex tube was sonicated then placed in a preheated oven for 48 h at 120 °C. The reaction mixture was then cooled down to room temperature and the solid crystalline material was filtered and washed two times with synthesis solvent mixture and twice with methanol. Then, it was dried in an oven at 80 °C overnight.

Zn-IRMOF-74 synthesized using 3 : 1 : 1 and 3 : 3 : 1 Zn(NO_3_)_2_·6H_2_O : l-Hyp : H_4_dobpdc ratios was prepared using a similar procedure.

#### Molecular simulations

High quality optimizations based on periodic unit cells using VASP 5.4.1 (parameters: ENCUT = 500, PREC = High, LAPSH = .true, EDIFF = 1e–7, EDIFFG = –2E–3).^39–42^ The used functional PBE + rVV1037 is a versatile van der Waals density functional developed by combining the PBE GGA functional with the rVV10 non–local correlation functional.^43,44^

## Conclusions

This work demonstrates that chiral inductors and synthesis solvents influence the crystallization of Zn-IRMOF-74 based on the strength and type of their interaction with the building-blocks of the porous molecular framework. Pyrrolidone-based solvents improve the thermal stability of the framework, especially in the case of NBP that has aromatic rings able to interact with the framework's walls. Alkaloids have weak interactions with Zn-IRMOF-74 and do not influence the crystallization process. In the case of *trans*-4-hydroxyproline as chiral additive, strong binding to the open metal ion sites through the carboxylate group is observed for a low concentration of additive. An increased concentration of 4-hydroxyproline leads to additional formation of [Zn(l-Hyp)_2_], thus decreasing the yield of the reaction and even preventing the crystallization of Zn-IRMOF-74. Molecular simulations show that 4-hydroxyproline can coordinate in a bidentate manner and influence the spatial arrangement around the metal ions. Furthermore, its coordination leads to changes in the orientation of the aromatic rings of the organic linker. These results provide insights into the complexity of chirality induction in porous molecular assembly and would benefit the development of highly efficient chirality control strategies.

## Conflicts of interest

There are no conflicts to declare.

## Supplementary Material

DT-050-D1DT01945G-s001
