# Correction: Role of additives and solvents in the synthesis of chiral isoreticular MOF-74 topologies

**DOI:** 10.1039/d1dt90146j

**Published:** 2021-08-31

**Authors:** Andreea Gheorghe, Suzanne Reus, Mark Koenis, David Dubbeldam, Sander Woutersen, Stefania Tanase

**Affiliations:** Van‘t Hoff Institute for Molecular Sciences, University of Amsterdam Science Park 904 1098 XH Amsterdam The Netherlands s.grecea@uva.nl

## Abstract

Correction for ‘Role of additives and solvents in the synthesis of chiral isoreticular MOF-74 topologies’ by Andreea Gheorghe *et al.*, *Dalton Trans.*, 2021, DOI: 10.1039/D1DT01945G.

The e-mail address of the corresponding author Stefania Tanase was given incorrectly; it should be s.grecea@uva.nl.

The correct author list and affiliations is as given above.

The Royal Society of Chemistry apologises for these errors and any consequent inconvenience to authors and readers.

## Supplementary Material

