# Supplementary material for: Role of additives and solvents in the synthesis of chiral isoreticular MOF-74 topologies
Source: Dalton Trans. 2021 Jul 21;50(35):12159–67. doi: 10.1039/d1dt01945g (PMC8439144; doi:10.1039/d1dt01945g)
Supplement: DT-050-D1DT01945G-s001 [file DT-050-D1DT01945G-s001.pdf]

Electronic Supplementary Material (ESI) for Dalton Transactions.
This journal is © The Royal Society of Chemistry 2021

Supplementary information

Role of additives and solvents in the synthesis of chiral isoreticular MOF-74 topologies

Andreea Gheorghe, Suzanne Reus, Mark Koenis, David Dubbeldam, Sander Woutersen and Stefania Tanase*

Van 't Hoff Institute for Molecular Sciences, University of Amsterdam, Science Park 904, 1098 XH, Amsterdam, The Netherlands.

*To whom correspondence should be addressed.

E-mail: **s.grecea@uva.nl**

S1. Ligand synthetic procedures

Dimethyl 3,3'–dimethoxy–[1,1'–biphenyl]–4,4'–dicarboxylate was synthesized following a modified literature procedure.[1] Methyl 4–iodo–2–methoxybenzoate (2.31 g, 7.91 mmol, 1 eq), 4–methoxycarbonyl–3–methoxyphenyl boronic acid (2.0 g, 9.5 mmol, 1.2 eq), K_2CO_3 (6.65 g, 48.1 mmol, 6.1 eq) and [1,1'-bis(diphenylphosphino)ferrocene]palladium(II) chloride (0.34 g, 0.46 mmol, 0.06 eq) were dissolved in a mixture of water (20 mL) and 1,4–dioxane (50 mL) that was previously degassed by bubbling nitrogen for 20 min. The reaction was refluxed for 17 h. The mixture was cooled down to room temperature and water (40 mL) was added. After filtration, a shiny grey solid was obtained and washed three times with water. The product was recrystallized from an excess of DMF (140 mL) and water (4 mL). A grey–white solid (1.63 g, 4.92 mmol, 62% yield) was obtained.

^{1}H–NMR (DMSO–d_6): δ = 7.76 (d, J = 8.0 Hz, 1H), 7.40 (dd + d, J = 8.0, 1.2 Hz, 2H), 3.94 (s, 3H), 3.81 (s, 3H) ppm. FTIR (KBr, cm^{-1}): 2998 (w, sh), 2950 (w, sh), 2840 (w, sh), 1700 (s, sh), 1602 (s, sh), 1553 (s, sh), 1462 (m, sh), 1435 (s, sh), 1392 (s, sh), 1312 (s, sh), 1251 (s), 1153 (s, sh), 1104 (s, sh), 1025 (s, sh), 964 (w), 836 (m, sh), 775 (s, sh). ESI–MS *m/z* (%): 330.109 (49), 299.090 (100), 297.074 (34), 225.054 (15), 139.054 (20).

3,3'–Dimethoxy–[1,1'–biphenyl]–4,4'–dicarboxylic acid was synthesized following a modified literature procedure.[2] Dimethyl 3,3'–dimethoxy–[1,1'–biphenyl]–4,4'–dicarboxylate (3.25 g, 9.84 mmol, 1 eq) and potassium hydroxide (3.31 g, 59.0 mmol, 6 eq) were added to a 500 mL round bottom flask containing methanol (115 mL). The mixture was refluxed for 1 h, after which it was cooled down to room temperature. Water (100 mL) was added and the solution was extracted three times with diethyl ether (1x 150 mL, 2x 70 mL). The solution was acidified using 6 M hydrochloric acid to form a white solid. The product (2.88 g, 9.53 mmol, 97% yield) was recovered through vacuum filtration, washed three times with water and dried overnight under vacuum at 70 °C.

^{1}H–NMR (DMSO–d_6): δ = 12.67 (s, 1H), 7.75 (d, J = 8.0 Hz, 1H), 7.38 (dd + d, J = 8.0, 1.2 Hz, 2H), 3.94 (s, 3H) ppm. FTIR (KBr, cm^{-1}): 3235 (m, br), 3000 (w, sh), 2958 (w, sh), 2849 (w, sh), 1732 (s, sh), 1606 (s, sh), 1557 (m, s), 1402 (s, sh), 1362 (s, br), 1237 (s, sh), 1188 (m, sh), 1139 (m, sh), 1012 (s, sh), 830 (m, sh), 766 (m, sh), 684 (m). ESI–MS *m/z* (%): 905.214 (40), 603.141 (100), 301.073 (67).

3,3'–dihydroxy–[1,1'–biphenyl]–4,4'–dicarboxylic acid (H_4dobpdc). Hydrogen bromide (33% in glacial acetic acid, 25 mL) was added to compound 3,3'–dimethoxy–[1,1'–biphenyl]–4,4'–dicarboxylic acid (1.64 g, 0.56 mmol, 1 eq) in a 50 mL round bottom flask. The mixture was heated slowly to 127 °C and then kept under reflux for 15 h. A double gas trap with 5M sodium hydroxide was used to neutralize the acidic fumes coming out of the flask. The mixture was cooled down to room temperature and the resulting solid filtered and washed three times with cold water, then dried overnight at 80 °C to obtain a white solid (1.16 g, 4.22 mmol, 78% yield).

^{1}H–NMR (DMSO–d_6): δ = 13.88 (s, 1H), 11.58 (s, 1H), 7.88 (m, 1H), 7.29 (m, 2H) ppm. ^{13}C–NMR (DMSO–d_6): [illegible] = 171.6, 161.3, 145.6, 130.9, 118.0, 115.2, 112.8 ppm. FTIR (KBr, cm^{-1}): 3002 (m, br), 2866 (m, br), 1660 (s, sh), 1620 (s, sh), 1457 (s, sh), 1435(s, sh), 1290 (s, sh), 1235 (s, sh), 967 (w, sh), 888 (w, sh), 863 (m, sh), 772 (m, sh), 695 (w, sh), 663 (w, sh), 479 (w, sh). ESI–MS *m/z* (%): 273.0402 (100).

S2. Synthesis of [Zn(*L-Hyp*)$_2$]

[Zn(*L-Hyp*)$_2$] was synthesized following the reported procedure for the synthesis of [Zn(*L–Pro*)$_2$].[3] NaOH (0.174 g, 4.34 mmol, 2.3 eq) and *L-Hyp* (0.57 g, 4.34 mmol, 2.3 eq) were dissolved in MeOH (10 mL). The solution was stirred for 10 min and $Zn(CH_3COO)_2 \cdot 2H_2O$ (0.476 g, 1.82 mmol, 1 eq) was added. The solution was stirred for another 35 min to form the white

precipitate. The solid was recovered by vacuum filtration and dried further in an oven at 70 °C. FTIR (KBr, cm^{-1}): 3340 (s, br), 3303 (s, sh), 3247 (s, sh), 3205 (w, br), 2979 (w, sh), 2965 (w, sh), 2946 (w, sh), 2913 (m, sh), 2890 (m, sh), 1623 (s, sh), 1596 (s, sh), 1446 (m, sh), 1419 (m, br), 1402 (m, br), 1340 (m, sh), 1315 (m, sh), 1268 (w, sh), 1240 (w, sh), 1213 (w, br), 1205 (w, br), 1191 (w, sh), 1097 (m, sh), 1083 (m, sh), 1062 (m, sh), 1049 (m, sh), 1041 (m, sh), 989 (w, sh), 958 (m, sh), 931 (m, sh), 910 (w, sh), 871 (w, sh), 863 (w, sh), 854 (w, sh), 800 (w, sh), 769 (w, sh), 763 (w, sh), 736 (w, sh), 719 (w, sh), 682 (w, br), 661 (w, br), 632 (w, br), 559 (m, sh), 495 (w, sh), 457 (w, sh). ESI-MS (positive) calc. for $[M+Na]^+$ ($C_{10}H_{16}N_2O_6ZnNa$) 347.0198, found 347.0218; $[2M+Na]^+$ ($C_{20}H_{32}N_4O_{12}Zn_2Na$) 671.0497, found 671.0327; $[3M+Na]^+$ ($C_{30}H_{48}N_6O_{18}Zn_3Na$) 995.0797, found 995.0510.

The synthesis of [Zn(*D-Hyp*)$_2$] required a 16h reaction time.

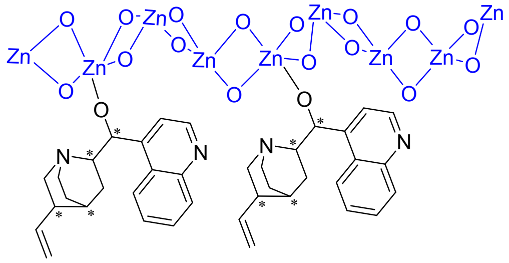

Figure S1. Proposed coordination of the cinchona alkaloid, using its deprotonated hydroxyl group, to the Zn^{2+} metal centres on the helical SBU of Zn-IRMOF-74 (blue). For clarity, alkaloid coordination was shown to only two consecutive open metal ions that are oriented into the same 1D channel.

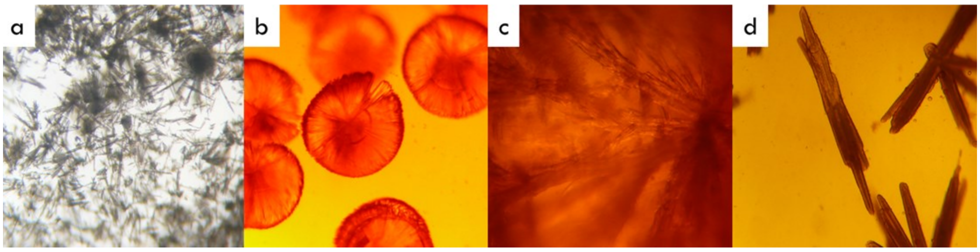

Figure S2. Polarised optical microscope images of the crystals obtained for the crystallisation of Zn-IRMOF-74 in DMF (a), NMP (b), CHP (c) and NBP (d).

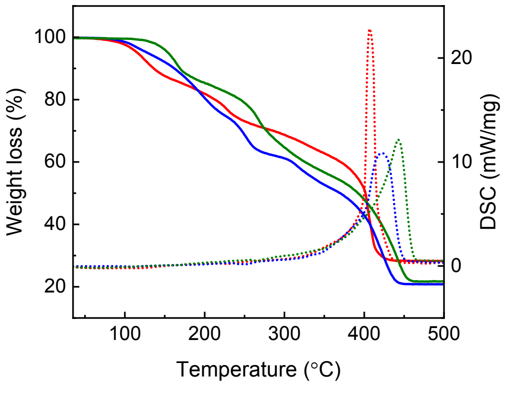

Figure S3. TGA-DSC curves of Zn-IRMOF-74 synthesised in DMF (red), NMP (blue) and NBP (green).

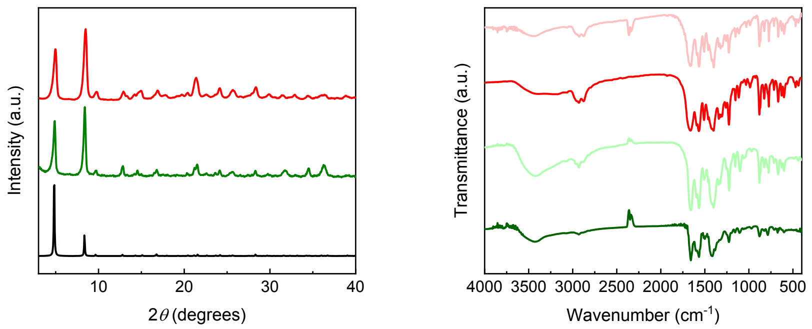

Figure S4. Left: PXRD patterns (left) of simulated Zn-IRMOF-74 (black), Zn-IRMOF-74 obtained in the presence of (-)-cinchonidine in DMF (green) and NMP (red). Right: FTIR of Zn-IRMOF-74 samples synthesised in DMF (light green) and NMP (light red), and of Zn-IRMOF-74 obtained in the presence of (-)-cinchonidine in DMF (green) and NMP (red).

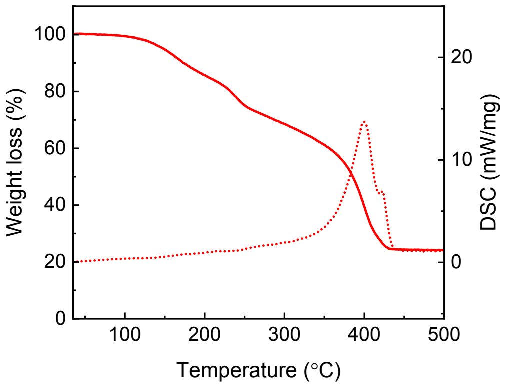

Figure S5. TGA-DSC curves of Zn-IRMOF-74 obtained in the presence of (-)-cinchonidine in NMP.

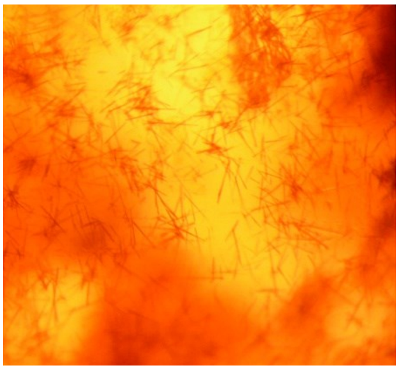

Figure S6. Zn-IRMOF-74 synthesised in the presence of (-)-cinchonidine in NMP.

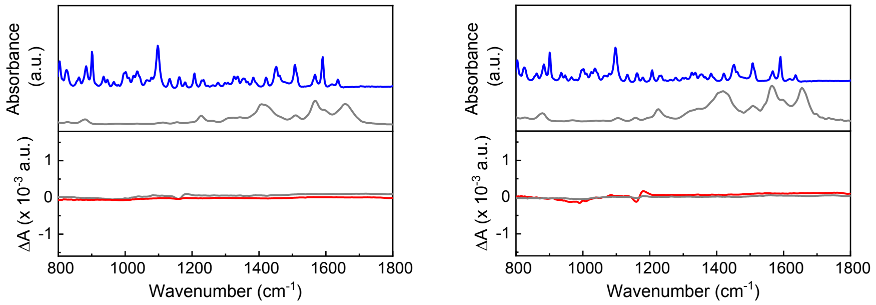

Figure S7. FTIR (top) of (-)-cinchonidine (blue) and Zn-IRMOF-74 synthesised in the presence of (-)-cinchonidine (grey) in NMP (left) or in DMF (right). VCD spectra (bottom) of Zn-IRMOF-74 synthesised in the presence of (+)-cinchonine (red) and (-)-cinchonidine (grey) in NMP (left) or

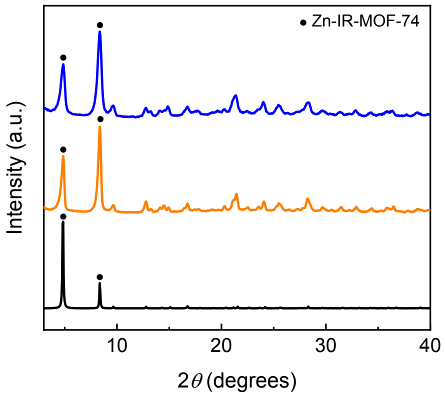

in DMF (right).

Figure S8. PXRD patterns of calculated Zn-IRMOF-74 (black), Zn-IRMOF-74 obtained in the presence of (+)-cinchonine in DMF (orange) and NMP (blue).

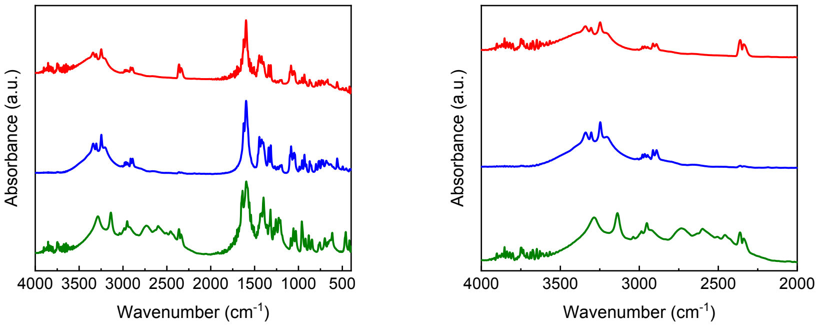

Figure S9. FTIR spectra of [Zn(*L-Hyp*)$_2$] (blue), [Zn(*D-Hyp*)$_2$] (red) and *L-Hyp* (green).

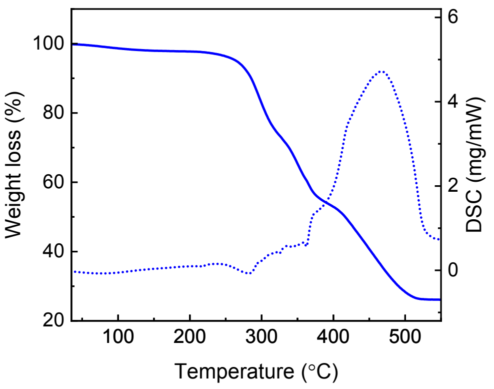

Figure S10. TGA-DSC curves of [Zn(*L-Hyp*)$_2$].

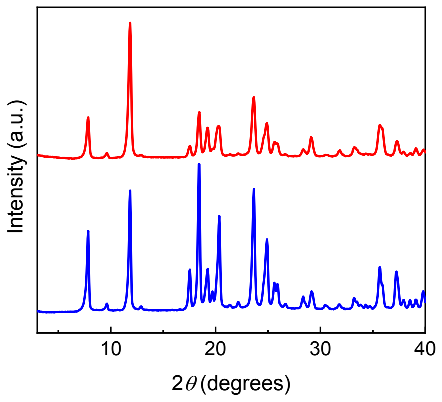

Figure S11. PXRD patterns of [Zn(*D-Hyp*)$_2$] (red) and [Zn(*L-Hyp*)$_2$] (blue).

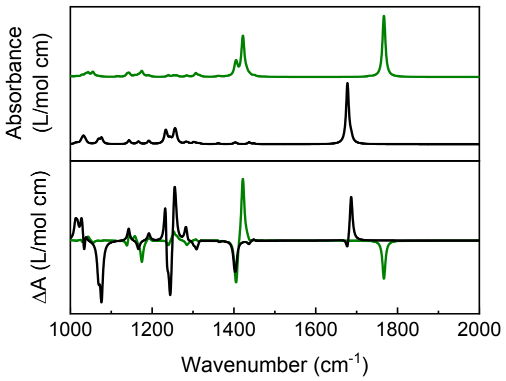

Figure S12. Calculated FTIR spectra (top) and calculated VCD spectra (bottom) of *L-Hyp* (green) and [Zn(*L-Hyp*)$_2$] (black), computed with DFT in ADF2019 at a TZP/BP86 level of theory.

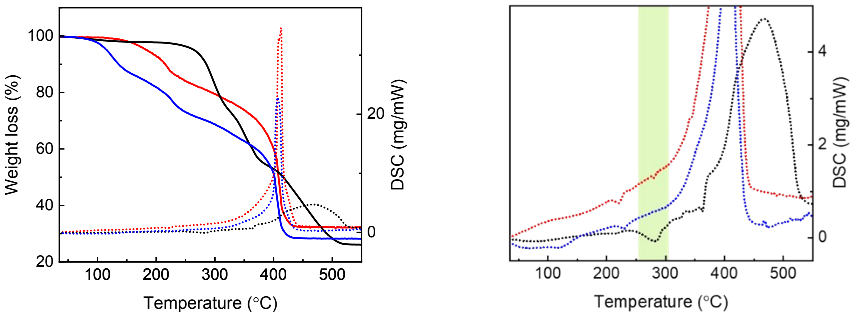

Figure S13. TGA–DSC (left) and DSC (right) curves of [Zn(*L-Hyp*)$_2$] (black), Zn-IRMOF-74 synthesised in DMF (blue) and Zn-IRMOF-74 synthesised using a Zn^{2+}: H_4dobpdc: *L-Hyp* ratio equal to 3:1:1 (red).

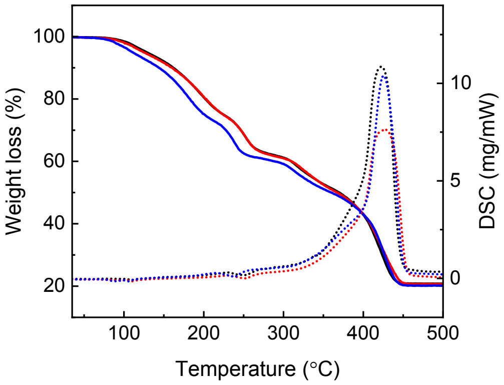

Figure S14. TGA–DSC curves of Zn-IRMOF-74 synthesized in NMP (black) and Zn-IRMOF-74 synthesised using a Zn^{2+}: H_4dobpdc: *Hyp* ratio equal to 3:1:1 with *L-Hyp* (red) or *D-Hyp* (blue).

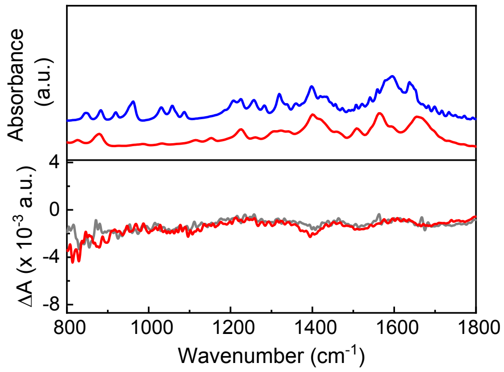

Figure S15. FTIR spectra (top) of *L-Hyp* (blue) and Zn-IRMOF-74 synthesised in NMP in the presence of *L-Hyp* (grey) and VCD spectra of Zn-IRMOF-74 synthesised in NMP in the presence of *L-Hyp* (grey) and *D-Hyp* (red).

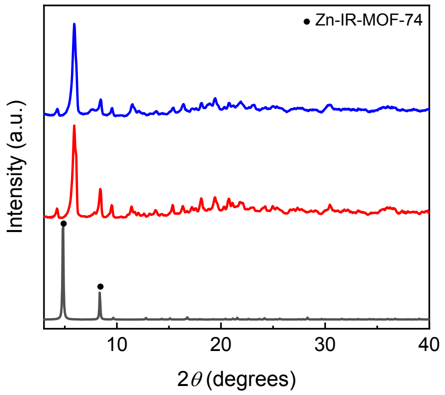

Figure S16. Calculated PXRD pattern of Zn-IRMOF-74 (black) and Zn-IRMOF-74 synthesized in NBP using a 3:1:1 Zn^{2+}: H_4dobpdc: *Hyp* ratio in the presence of *L-Hyp* (grey) and *D-Hyp* (red).

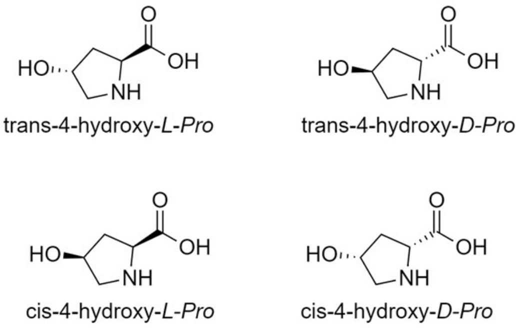

Figure S17. Molecular structures of the stereoisomers of 4-hydroxyproline.

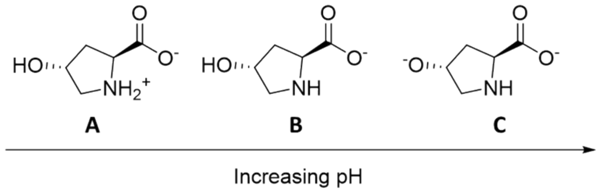

Figure S18. Molecular structures of *L-Hyp* for different pH values.

Table S1. The binding energies for the different structures (**A**-zwitterion, **B**-deprotonated carboxylate, and **C**-deprotonated carboxylate and hydroxyl group) of trans-4-hydroxy-*L-Pro*, trans-4-hydroxy-*D-Pro*, cis-4-hydroxy-*L-Pro* and cis-4-hydroxy-*D-Pro* with Zn-IRMOF-74 *via* carboxylate oxygens. The different values of the binding energies correspond to different orientations.

Chiral additive	Binding energy for binding via O (kJmol^{-1})							
	A			B				C[a]
trans-4-hydroxy-*L-proline* (*L-Hyp*)	-215.94	-254.64	-223.49	-225.90	-203.39	-419.55	-441.17	-269.98
trans-4-hydroxy-*D-proline* (*D-Hyp*)	-276.79	-262.49		-261.91	-235.16	-428.11	-424.04-	-210.45
cis-4-hydroxy- *L-proline*	-175.09	-249.02		-242.96	-178.74	-465.03	-186.93	-170.13
cis-4-hydroxy-*D-proline*	-199.87	-152.16		-230.25	-389.58	-536.97		-202.50

a. Structure of the additive is not preserved (ring opening) or structure is rearranged (ring opening and closing).

trans-4-hydroxy-*D*- *proline* (*D-Hyp*)
A

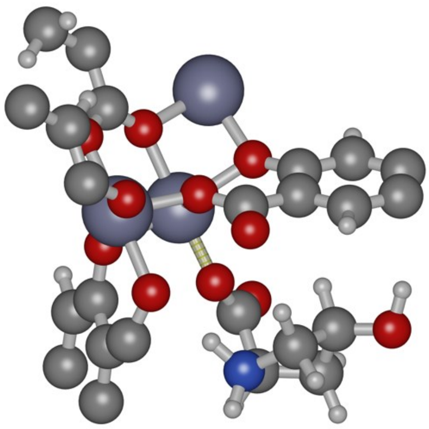

trans-4-hydroxy-*L*- *proline* (*L-Hyp*)
A

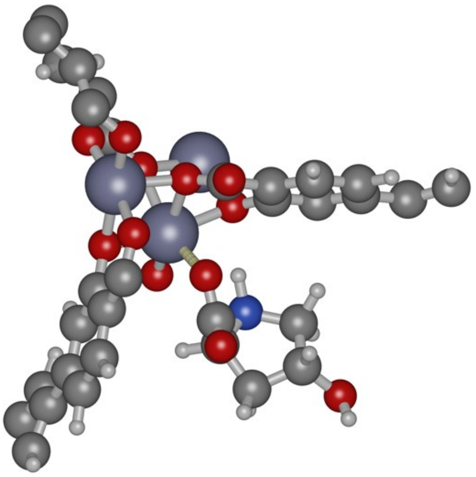

trans-4-hydroxy-*D*- *proline* (*D-Hyp*)
B

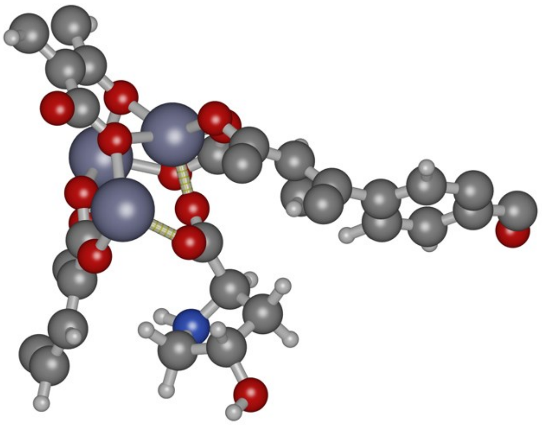

trans-4-hydroxy-*L*- *proline* (*L-Hyp*)
B

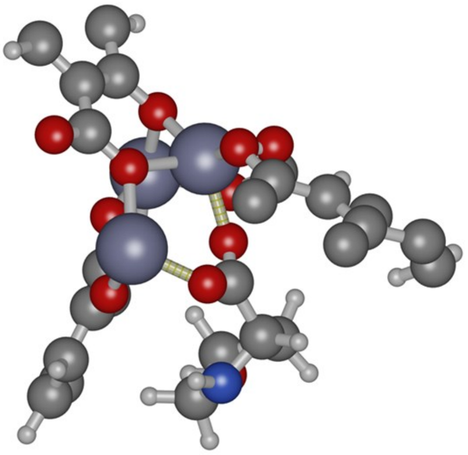

trans-4-hydroxy-*D*- *proline* (*D-Hyp*)
C

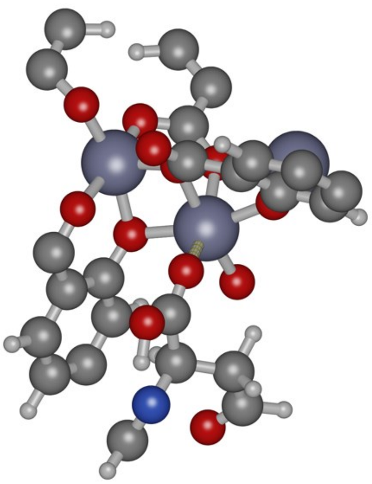

trans-4-hydroxy-*L*- *proline* (*L-Hyp*)
C

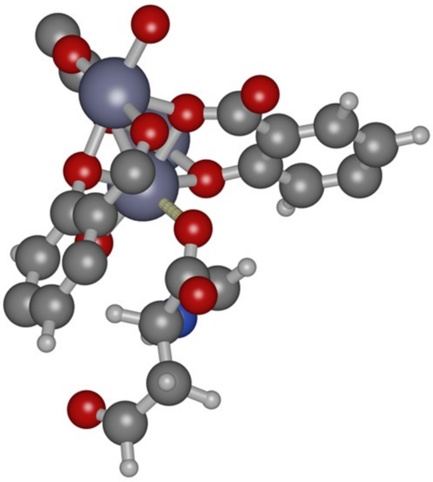

Figure S19. Binding representations of *trans* isomers of 4-hydroxyproline with Zn^{2+} metal ions of Zn-IRMOF-74.

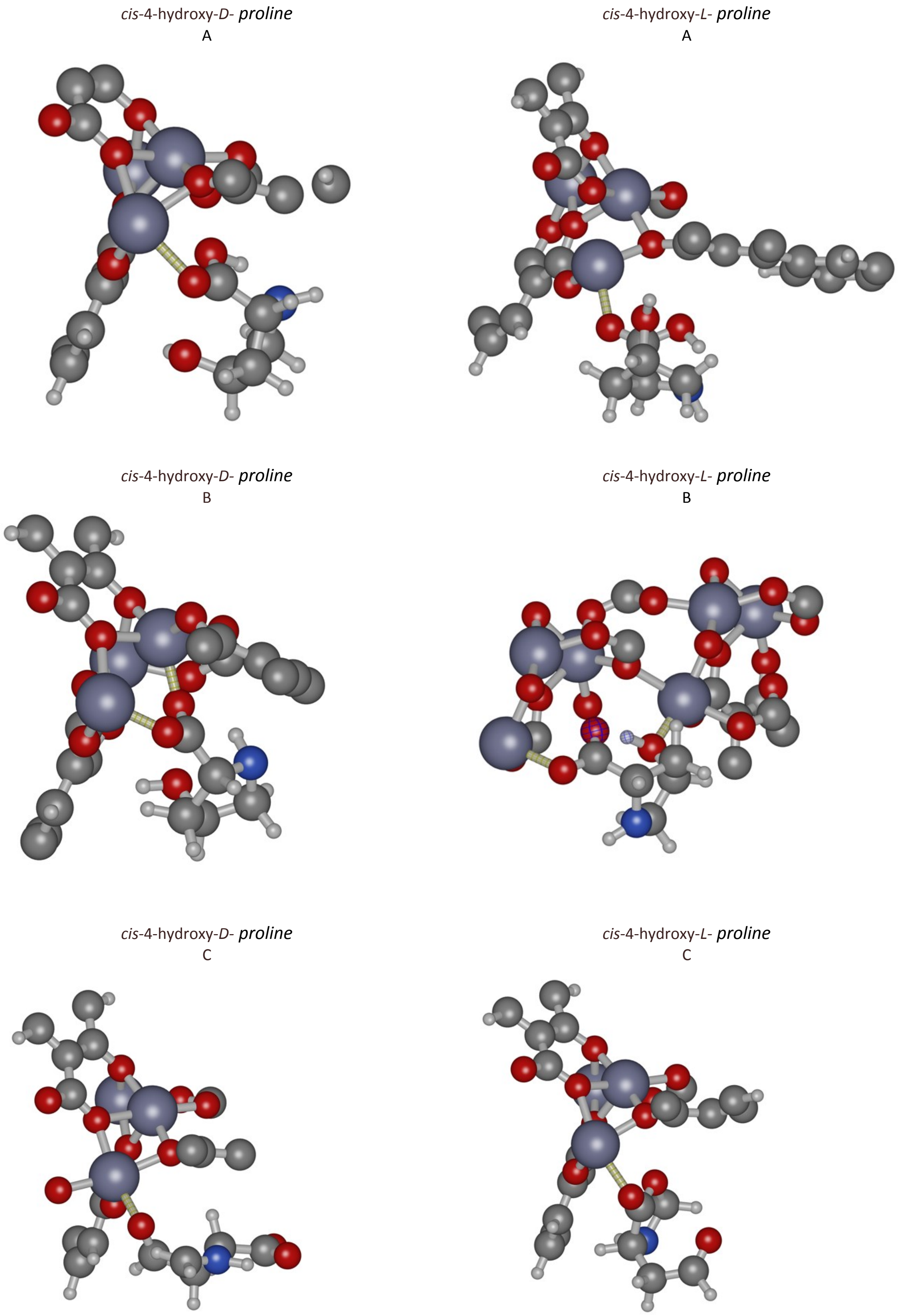

Figure S20. Binding representations of *cis* isomers of 4-hydroxyproline with Zn^{2+} metal ions of Zn-IRMOF-74.

Table S2. Properties of the O-H···O intramolecular bond in the *cis*-4-hydroxy-*L-proline* upon binding to Zn^{2+}metal ions of Zn-IRMOF-74 compared to literature reported properties for strong H bonds.[4]

Property	Ref. 4	Calculated
Bond lengths (Å)	H···O > O-H	1.501 > 1.039
Lengthening of O-H (Å)	0.01-0.05	0.097
O…O (Å)	2.5-3.2	2.54
H···O (Å)	1.5-2.2	1.501
O-H···O bond angle (°)	130-180	164.39

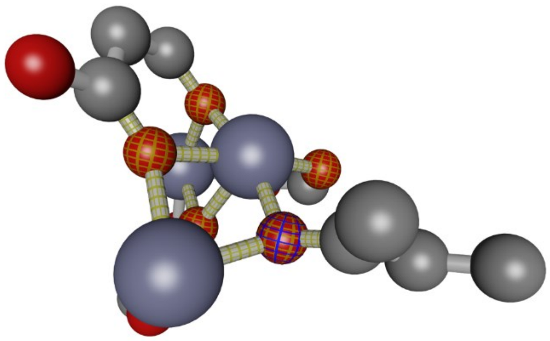

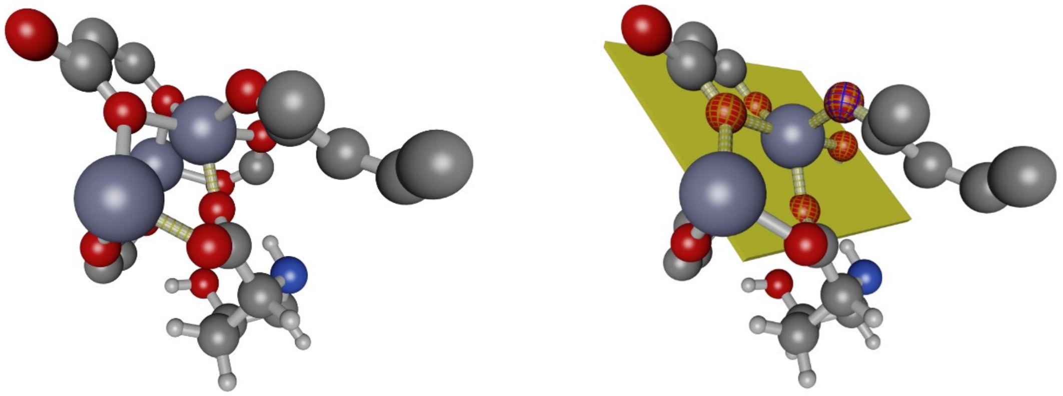

Figure S21. Top: coordination geometry of pentacoordinated Zn^{2+} metal ions of Zn-IRMOF-74.Bottom: changes due to the bidentate coordination of *cis*-4-hydroxy-*D*-proline (deprotonated carboxylic form B) via the carboxylate oxygens to consecutive Zn^{2+} metal ions of Zn-IRMOF-74. The yellow plane denotes a reference plane.

References

1 G. E. M. Schukraft, S. Ayala, B. L. Dick and S. M. Cohen, *Chem. Commun.*, 2017, **53**, 10684–10687.
2 X.-Z. Wang, D.-R. Zhu, Y. Xu, J. Yang, X. Shen, J. Zhou, N. Fei, X.-K. Ke and L.-M. Peng, *Cryst. Growth Des.*, 2010, **10**, 887–894.
3 M. P. D. Rocha, A. R. Oliveira, T. B. Albuquerque, C. D. G. da Silva, R. Katla and N. L. C. Domingues, *RSC Adv.*, 2016, **6**, 4979–4982.
4 J. M. MacLeod and F. Rosei, in *Comprehensive Nanoscience and Technology*, eds. D. L. Andrews, G. D. Scholes and G. P. Wiederrecht, Academic Press, Amsterdam, 2011, p. 15.
